# Medical cannabinoids for treatment of neuropsychiatric symptoms in dementia: a systematic review

**DOI:** 10.47626/2237-6089-2021-0288

**Published:** 2021-12-10

**Authors:** Florindo Stella, Leandro C. Lane Valiengo, Vanessa J. R. de Paula, Carlos Augusto de Mendonça Lima, Orestes V. Forlenza

**Affiliations:** 1 Departamento e Instituto de Psiquiatria HCFMUSP Faculdade de Medicina Universidade de São Paulo São Paulo SP Brazil Laboratory of Neuroscience (LIM-27), Departamento e Instituto de Psiquiatria HCFMUSP, Faculdade de Medicina, Universidade de São Paulo, São Paulo, SP, Brazil.; 2 Instituto Nacional de Biomarcadores em Neuropsiquiatria (InBion) Conselho Nacional de Desenvolvimento Científico e Tecnológico São Paulo SP Brazil Instituto Nacional de Biomarcadores em Neuropsiquiatria (InBion), Conselho Nacional de Desenvolvimento Científico e Tecnológico, São Paulo, SP, Brazil.; 3 Instituto de Biociências Universidade Estadual Paulista SP Brazil Instituto de Biociências, Universidade Estadual Paulista (UNESP), Campus de Rio Claro, SP, Brazil.; 4 World Psychiatric Association Geneva Switzerland Section of Old Age Psychiatry, World Psychiatric Association, Geneva, Switzerland.

**Keywords:** Dementia, neuropsychiatric symptoms, cannabidiol, Δ^9^-tetrahydrocannabinol, treatment

## Abstract

**Introduction:**

Neuropsychiatric symptoms are an integral component of the natural history of dementia, occurring from prodromal to advanced stages of the disease process and causing increased burden and morbidity. Clinical presentations are pleomorphic and clinical management often requires combinations of pharmacological and non-pharmacological interventions. However, limited efficacy and a non-negligible incidence of adverse psychotropic drug events emphasize the need for novel therapeutic options.

**Objectives:**

To review the evidence supporting use of medical cannabinoids for treatment of neuropsychiatric symptoms (NPS) of dementia.

**Methods:**

We conducted a systematic review of the medical literature to examine scientific publications reporting use of medical cannabinoids for treatment of NPS. Medical Subject Headings (MeSH) were used to search for relevant publications and only papers reporting original clinical information were included. A secondary search was performed within selected publications to capture relevant citations that were not retrieved by the systematic review. The papers selected were categorized according to the level of evidence generated by the studies in relation to this clinical application, i.e. (1) controlled clinical trials; (2) open-label or observational studies; and (3) case reports.

**Results:**

Fifteen publications with original clinical data were retrieved: five controlled clinical trials, three open-label/observational studies, and seven case reports. Most studies indicated that use of medical cannabinoids engendered favorable outcomes for treatment of NPS related to moderate and advanced stages of dementia, particularly agitation, aggressive behavior, sleep disorder, and sexual disinhibition.

**Conclusion:**

Medical cannabinoids constitute a promising pharmacological approach to treatment of NPS with preliminary evidence of benefit in at least moderate to severe dementia. Controlled trials with longitudinal designs and larger samples are required to examine the long-term efficacy of these drugs in different types and stages of dementia, in addition to their adverse events and risk of interactions with other drugs. Many pharmacological details are yet to be determined, such as dosing, treatment duration, and concentrations of active compounds (e.g., cannabidiol [CBD]/ Δ^9^-tetrahydrocannabinol [THC] ratio) in commercial preparations of medical cannabinoids.

## Introduction

Neuropsychiatric symptoms (NPS) are relevant clinical manifestations that pertain to the natural history of dementia, with prevalence estimates ranging from 35 to 95% depending on the severity of clinical deterioration.^1 - 3^ These manifestations are also referred to as behavioral and psychological symptoms of dementia (BPSD), and may present with severe and disabling behaviors, critically impacting on the well-being of both patients and caregivers. NPS are ubiquitous across different etiologies of dementia disorders, but particularly important in Alzheimer’s disease (AD), frontotemporal dementia (FTD), Lewy body dementia (LBD), and other forms of dementia with Parkinsonism. In AD, for instance, NPS can be observed from the pre-dementia phase through all stages of dementia, with increasing incidence and magnitude as the disease progresses.^4 , 5^ At prodromal and preclinical stages of AD, the occurrence of NPS is associated with increased risk of conversion to dementia and subsequent cognitive and functional deterioration.^6^ In most etiologies, NPS tend to become more prevalent with progression of the neurodegenerative processes, in which case symptoms and abnormal behaviors may illustrate the damage to specific brain areas.^2 , 3^ Relevant and persistent behavioral abnormalities have been related to increased morbidity and higher mortality risk. As a general rule, the occurrence of persistent symptoms often accounts for debilitating outcomes and predicts more severe global deterioration.^2^ Also, NPS invariably increase caregiver burden, being associated with early nursing home placement and hastening the decision to institutionalize.^7^

Effective treatment of NPS is still a challenge in clinical practice. At earlier stages of dementia in AD and related disorders, regular use of cholinesterase inhibitors and meantime in therapeutic doses is recommended as the first step in the pharmacological management of mild behavioral changes. However, in the presence of severe and disruptive behaviors such as psychosis and agitation, which often occur in advanced stages of dementia, other psychotropic drugs must be considered, including antipsychotics, antidepressants, benzodiazepines, and anticonvulsants. Nonetheless, studies of psychopharmacological interventions for treatment of NPS have so far yielded at best modest evidence of efficacy, which must be counterbalanced by relevant safety concerns. Special attention must be given to increased risk of cerebrovascular events and all-cause mortality, especially when these medications are used for extended periods.^8 - 10^ On the other hand, non-pharmacological strategies, although more acceptable and safer, may not be efficacious (or even inapplicable) in certain types of NPS, particularly the most severe. Nonetheless, it is widely accepted that these interventions must accompany and if possible precede introduction of psychotropic drugs for clinical management of NPS, with recognizable benefits for general state of health.^11^ In this context, new therapeutic options to treat NPS are needed and eagerly awaited by the clinical and scientific community.

The endocannabinoid system (eCB) plays important homeostatic roles, modulating multiple physiological functions through signaling from widespread receptors (CB1 and CB2) present in the peripheral and central nervous systems. Systemically, the eCB is relevant to cardiovascular, immunological, and reproductive functions.^12^ In the brain, with signaling predominantly through CB1 receptors, it impacts on distinct neurobehavioral functions that range from cognition (learning and memory) to behavior (mood regulation, emotional control, appetite, feeding, and addictive behavior), in addition to sensitivity to pain and mechanisms of neuroprotection.^13^ Expression of CB1 receptors in the basal ganglia regulates locomotor activity,^14^ whereas their expression in the cortex and hippocampus is predominantly related to cognitive-behavioral effects as well as to psychotropic and antiepileptic properties. The ubiquitous expression of cannabinoid receptors in the brain also illustrates their potential to interact with other neurotransmitter systems, such as those mediated by acetylcholine, dopamine, GABA, histamine, serotonin, glutamate, norepinephrine, prostaglandins, and opioid peptides, explaining the pleomorphic pharmacological effects of cannabinoid drugs.

Pharmacological compounds that modulate the eCB have been more extensively tested in certain neurological and medical conditions, such as Parkinson’s disease, epilepsy, chronic pain, and incoercible nausea/vomiting secondary to cancer treatments.^15^ Expert reviews further suggest that use of these drugs may also be justified for treatment of autism, bipolar disorder, schizophrenia, Tourette syndrome, and depression, although construction of scientific evidence to support such indications is still in progress.^15^ Use of medical cannabinoids for management of NPS has attracted the attention of both clinicians and researchers in the recent past, envisaging the potential benefits of cannabidiol (CBD), Δ^9^ -tetrahydrocannabinol (THC), and combined preparations for management of difficult clinical situations. Preliminary and uncontrolled communications reported positive effects on severe and refractory symptoms that were otherwise unmanageable, particularly in cases of advanced dementia.^16^ However, the body of evidence to support use and to guide the prescription of medical cannabinoids for the treatment of NPS is still controversial. Therefore, the objective of the present study is to systematically review the medical literature to examine studies reporting on use of medical cannabinoids for treatment of NPS and to compile the available data on efficacy, tolerability, and dose regimens.

## Methods

We conducted a systematic and critical analysis of the specialized literature, searching for articles containing original data published in English in the PubMed database, using Medical Subject Headings (MeSH) terms. Thus, the following inclusion criteria guided the search strategy: (cannabinoid* or cannabidiol or CBD or Δ9-tetrahydrocannabinol or THC or endocannabinoid* or marijuana or nabilone or dronabinol) and (dementia or “cognitive impairment” or “cognitive decline” or Alzheimer* or “vascular dementia” or “frontotemporal dementia” or “semantic dementia” or aphasia or “language dementia” or “Lewy bodies” or “dementia in psychiatric diseases”) and (“behavioral and psychological symptoms of dementia” or BPSD or “neuropsychiatric symptoms” or agitation or aggression or delusions or hallucinations or depression or anxiety or irritability or apathy or “sleep disorders” or insomnia or “appetite disorder” or disinhibition or cognition or cognitive). We also performed a manual search for relevant citations identified in the selected articles that were not retrieved by the systematic search. Exclusion criteria comprised studies in which psychopathological manifestations were not the main target of treatment with medical cannabinoids, and those whose NPS did not arise from a dementia condition or were not clearly defined. To identify additional references that may have been missed by the systematic review, we conducted an active search of the citations from selected articles. Review articles, editorials, points of view, study protocols, and secondary analyses were ruled out, i.e., only papers reporting original clinical information were included in the analysis. We established three categories to rank publications according to the respective level of evidence provided on the efficacy of these compounds for treatment of NPS: controlled clinical trials with standardized NPS assessment (evidence level 1); open-label or observational studies with standardized NPS assessment (level 2); and case reports with or without standardized NPS assessment (level 3). The search covered a period extending from the 1990s to 28th February, 2021. Two researchers extracted the data from the reports (FS and LCLV).

We further analyzed the data according to clusters of target symptoms: agitation/aggression (including wandering, erratic motor behavior, threatening behavior, aggressive behavior); sleep disturbances (insomnia, nighttime behavior); and abnormal sexual behavior (sexual disinhibition, inappropriate sexual comments, public masturbation, touching).

## Results

**Figure 1:**
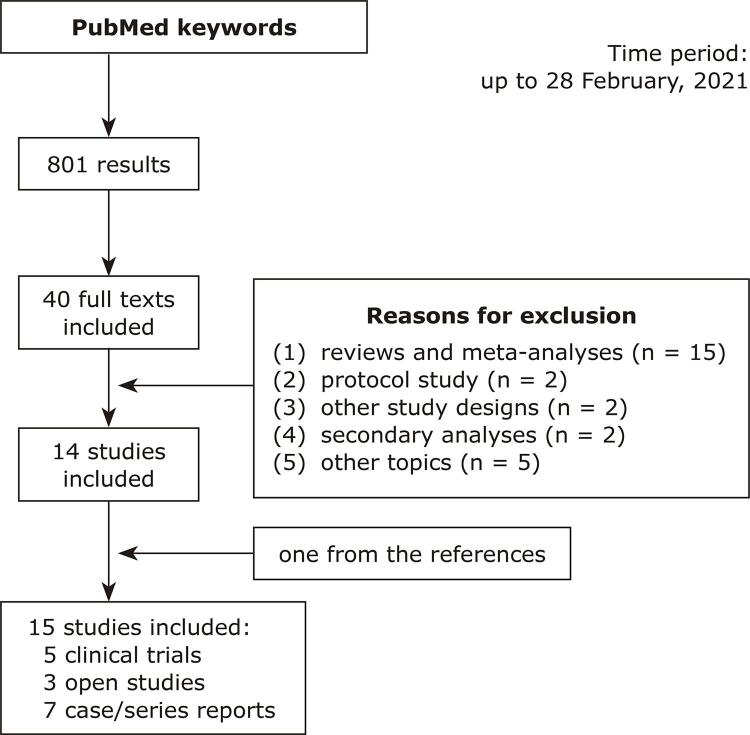
Schematic flow diagram illustrating literature search, articles excluded, and articles included in the review.

The search strategy yielded a total of 801 publications. Most of these did not meet the inclusion criteria, resulting in only 15 studies that reported original, clinical data ( Figure 1 ). Of these, five studies were controlled trials^17 - 21^ , three were open-label/observational studies, and seven were case reports.^16 , 22 - 30^ To facilitate description of the findings, the publications selected were grouped in the following subsections, according to the neuropsychiatric domains targeted by the intervention: (a) agitation; (b) sleep disturbances; (c) abnormal sexual behavior.

### Agitation

#### Controlled clinical trials

In a placebo-controlled study with a crossover design, Volicer et al.^17^ investigated the effect of dronabinol (an orexigenic THC formulation) on a fixed dose schedule (2.5 mg/day) for management of food refusal and behavioral disturbances in 15 patients with AD, most suffering from severe dementia.^15^ Dronabinol was prescribed for 6 weeks, followed by placebo for a similar period. Eleven patients completed the study. Treatment with dronabinol substantially improved anorexia and weight gain, and also reduced the severity of behavioral disorders, e.g., agitation and euphoria. Somnolence and tiredness were the most commonly reported adverse reactions, although not requiring the intervention to be discontinued. However, one patient developed a generalized seizure, an adverse event that was regarded as possibly related to the drug.

Mahlberg et al.^18^ examined two pharmacological compounds for treatment of patients with severe dementia due to AD (mean age 79.0 years) suffering from agitation, specifically nocturnal behavior. The authors prescribed dronabinol 2.5 mg daily for seven patients and melatonin 3.0 mg per day for another seven patients and compared these therapies over a 2-week period to 10 patients of similar age who received placebo. Nocturnal behavior was measured by means of actigraphy. Patients in both intervention groups (dronabinol or melatonin) showed a significant improvement in nocturnal agitation as compared to patients in the placebo group. However, an important methodological limitation concerns the aggregation of dronabinol and melatonin patients in the same group, limiting the assessment of the efficacy of dronabinol. Safety and tolerability were not described, which constitutes another limitation.

In a small randomized, controlled trial, van den Elsen et al.^19^ evaluated the effect of low doses of oral THC for treatment of neuropsychiatric manifestations for 12 weeks in a test group of 22 patients (mean age 76.4 years) with AD, vascular dementia (VD), or mixed dementia (AD/VD) presenting with severe NPS.^19^ Initially, patients received a low THC dosage of 0.75 mg twice daily, which was subsequently shifted to placebo. Thereafter, the THC dosage was increased to 1.5 mg twice a day and, again, was substituted with placebo. There were no advantages of THC compared to placebo in the measurement of NPS, including agitation and aggression. Nonetheless, low-dose THC was well tolerated and safe, supporting additional studies with escalating doses.

The same group conducted a randomized, double-blind, placebo-controlled trial to examine the efficacy and safety of low-dose oral THC (4.5 mg/day) for 3 weeks for treatment of NPS in patients with severe AD, VD, or AD/VD.^20^ The intervention group (n = 24, mean age 79.0 years) received dronabinol (1.5 mg t.i.d. [ *ter in die* , three times a day]), while the comparison group (n = 26, mean age 78.0 years) received placebo. Primary outcomes were negative, given that treatment with dronabinol did not reduce NPS in this study group. Nevertheless, the THC compound was well tolerated and patients had no clinically relevant side effects on vital signs, weight, or episodic memory.

Herrmann et al.^21^ conducted a randomized, double-blind, crossover trial with nabilone, a THC-analogue, synthetic cannabinoid that is used therapeutically for its antiemetic, orexigenic, and analgesic properties. Thirty-eight patients with moderate and severe AD (mean age 87.0 years) and treatment-resistant agitation were given 1.0-2.0 mg of nabilone daily for 14 weeks. The first part of the intervention consisted of a 1-week placebo phase followed by 6 weeks of nabilone treatment. The second part of the study again started with a placebo intervention for 1 week, followed by 6 additional weeks of treatment with the active drug. Nabilone treatment was associated with a significant decrease in agitation, besides important reduction in related caregiver burden. Sedation constituted the most common emerging adverse event, which deserves particular consideration.

#### Open-label and observational studies

In an open-label pilot study, Walther et al.^22^ examined the effects of dronabinol for six patients, aged 81.5 years (five with AD and one with VD at advanced stages of dementia) who suffered from behavioral and day-night rhythm disturbances. Patients received 2.5 mg of dronabinol daily for 2 weeks. Nighttime behavior was assessed using actigraphy. With this treatment, agitation and nocturnal motor activity decreased from baseline in all patients. Furthermore, irritability, anxiety and appetite disorders also improved. However, delusions, hallucinations, and apathy did not change during dronabinol therapy. The drug was well tolerated, with no reported adverse events during the study.

Ten consecutive inpatients (mean age 73.2 years) suffering from moderate to severe AD dementia completed a prospective open-label trial, conducted for a 4-week period to examine medical cannabis oil (MCO) for treatment of behavioral disturbances, agitation and aggression.^23^ The MCO contained THC extract from the Cannabis plant. Chemical analysis of the MCO signalized very low levels of some other phytocannabinoids, e.g., CBD: 0.05%; cannabichromes: 0.05%; cannabinol: 0.17%; tetrahydrocannabivarin: 0.02%; and tetrahydrocannabinolic acid: < 0.01%. The patients received MCO twice a day for 4 weeks, each dose initially containing 2.5 mg of THC. On the 3rd day the dosage was increased two-fold to the equivalent of 5.0 mg of THC b.i.d. ( *bis in die* , twice a day), and subsequently to 7.5 mg b.i.d. in the absence of side effects or safety concerns. The authors observed a significant decrease in agitation and aggression, but also in aberrant motor behavior, delusions, sleep disorder and nighttime behavior, irritability, and apathy. Additionally, caregiver distress was reduced. Three patients presented clinically relevant side effects: dysphagia (presumably unrelated to the treatment); recurrent falls (which were also present prior to the treatment); and confusion (which improved with reduction of the experimental drug from 5.0 mg to 2.5 mg b.i.d.).

In an observational study, Broers et al.^24^ examined the clinical efficacy and administration feasibility of a THC/CBD-based medication for management of NPS in a nursing home. They carried out a pilot study with 10 women suffering from severe dementia caused by different etiologies (AD, VD, and AD/VD) and presenting with serious behavioral disturbances and some motor rigidity. Patients received oral doses of the THC/CBD preparation, with acceptable tolerability. During the first 2 weeks of the trial, the average daily dosage prescribed to patients was 7.6 mg/13.2 mg of THC/CBD respectively, with titration up to 9.0 mg/18.0 mg over the following 2 months, with stable doses thereafter. The lowest daily dosage was 7.0 mg of THC and 14.0 mg of CBD, and the highest dosage was 13.0 mg and 26.0 mg, respectively. The authors reported benefits in overall behavioral disturbances and motor rigidity. Likewise, the staff members reported benefits in engagement with daily activities and reduction of troublesome behaviors (such as aggression, screaming, tearing clothes) in most patients, who appeared to be calmer, more relaxed, less irritable, and smiling more.

#### Case reports

Favorable outcomes from the experience with medical cannabinoids were also described in case reports. Passmore^16^ described the case of a 72 year-old man with severe AD who was transferred from his house to a nursing home because of persistent motor hyperactivity, disinhibition (indiscriminate disrobing and toileting), and verbal physical aggression towards co-residents and staff, and who was unresponsive to routine pharmacological approaches. After treatment with nabilone 0.5 mg b.i.d., he experienced a prompt and sustained reduction in the severity of agitation and aggression, with no emergent adverse events, and was discharged from the nursing home. His NPS remained controlled over the following 3 months.

Another interesting report, published by Amanullah et al.,^26^ describes the case of a 79-year old man diagnosed with advanced dementia due to AD presenting with increased agitation and caregiver burden, which caused nursing home placement. Upon admission, the patient was undergoing regular pharmacological treatment with donepezil, memantine, and antipsychotics with poor response in relation to the occurrence of NPS. The patient was medicated with haloperidol and quetiapine, but subsequently developed an episode of delirium that resolved after withdrawal of these psychotropics, with persistence of agitation and aggressive behavior. Thereafter, nabilone 0.5 mg was started and titrated to 0.5 mg t.i.d., resulting in long-lasting improvement of agitation and communication, as reported by the staff, who observed that the patient recovered a minimum ability to express himself using short sentences, became acquiescent to verbal command, and displayed emotional reactions, more frequent smiles, and increased eye contact. In the same publication, the authors also reported the case of a 60-year-old man with severe FTD with aggressive behavior and sleep disorders, requiring three-to-four employees for daily hospice care. The patient was on a regular psychotropic regimen with no benefits. Nabilone 0.5 mg b.i.d. was prescribed in association with zopiclone at bedtime, with substantial decrease in aggressiveness, and reduction (to one) of the number of staff members needed to provide daily care. The patient also began to express himself in single words and short sentences. Daily doses of nabilone were increased to 0.5 mg t.i.d. during follow-up due to relapse of aggressive behavior, along with increments in quetiapine up to 300 mg/day. Under this regimen, the patient became progressively less agitated and more cooperative with provision of personal care. The authors concluded that use of nabilone in combination with other medications provoked a sustained reduction of agitation and aggressive behavior.

Defrancesco and Hofer^29^ published another case report on dronabinol for treatment of NPS in a 79-year-old woman with severe AD. The patient was unresponsive to conventional psychopharmacological intervention, leading to polypharmacy, which in turn caused sedation and other relevant adverse effects. She underwent a progressive worsening of agitation, aggression, anxiety, and disruptive behavior. Dronabinol (magistral prescription of THC) was started at low dose and titrated to THC equivalents of 4.9-6.7 mg/day, with a considerable reduction of symptoms. Her emotional state improved, allowing nursing care without anxious or aggressive behavior, without side effects. The patient maintained the improvement for at least 10 months, until the current report was written, with a low dosage of dronabinol and optimization of other psychotropics at lower doses.

In order to treat a male inpatient with LBD who presented with severe outbursts of aggression with violence against other patients and staff members, Wilhelm et al.^28^ prescribed dronabinol in progressive dosages, starting from 2.5 mg to 15.0 mg per day since conventional drugs had no efficacy. At lower prescriptions, there was no favorable effect, and with increasing doses, the agitation worsened. The treatment was changed to 24.0 mg dextromethorphan and 10.0 mg paroxetine, and after 1 day the patient showed a substantial improvement in aggressive behavior. Unfortunately, the patient killed himself by hanging 6 weeks after referral, but the authors argued that this event was not clearly associated with current medications.

Walther et al.^25^ described two treatment approaches with dronabinol for NPS: a 75-year-old man (patient A) and an 81-year-old man (patient B) with moderate AD presenting with circadian rhythm disorders, verbal agitation, and aggression. The patients were prescribed either dronabinol 2.5 mg/day for the first 2 weeks and placebo for the following 2 weeks (patient A), or the other way round (patient B). In the former case, nocturnal motor activity consistently decreased until the 3rd week of treatment, but symptoms returned to the baseline levels at the end of the placebo phase. As for patient B, who received placebo in the first 2 weeks and dronabinol in weeks 3 and 4, nocturnal motor activity was unchanged during the placebo phase and attenuated in the 1st week of dronabinol treatment, relapsing again in the 2nd week of dronabinol. Both patients tolerated the dronabinol treatment well, with no occurrences of severe adverse events.

#### Sleep disturbances

##### Case report

Effects of dronabinol on sleep disturbances were also reported for patients A and B in the aforementioned study by Walther et al.^25^ Both patients’ circadian rhythms were strengthened and there was a short-lived effect of dronabinol reducing nighttime activity for patient B, i.e., the effect waned after 1 week of treatment. The authors suggested that higher doses of dronabinol might be needed for a sustained improvement of nocturnal behavioral disturbances. The beneficial effect of dronabinol on sleep regulation is presumably related to modulation of the eCB by stimulation of CB1 receptors in the brainstem and subsequent effects on circadian rhythms.^31^

## Abnormal sexual behavior

### Case report

Zajac et al.^27^ report the case of man with mixed vascular and frontotemporal dementia living in a long-term assisted residence who exhibited sexually disinhibited behaviors, such as inappropriate sexual comments, public masturbation, touching the nursing staff, and harassing female residents. Non-pharmacological interventions and several interventions with psychotropic drugs were ineffective.^27^ The patient was prescribed nabilone 0.5 to 1.0 mg b.i.d. for 2 weeks and risperidone (0.5 mg orally) when indispensable to control aggressive behavior. The patient achieved a significant improvement in behavioral changes, with complete resolution of sexual disinhibition symptoms. During the treatment, the patient experienced some acute episodes of sedation and lethargy attributed to interaction of nabilone with other sedative drugs, or to a common adverse effect of the medication. When nabilone treatment was eventually interrupted, inappropriate sexual behaviors recurred, but were controlled again with reintroduction of the medication (0.5 mg t.i.d.) and there were continuous gains along the following 3 months of observation.

The main characteristics of the studies selected are presented in Table 1 .

**Table 1:** Intervention studies of medical cannabinoids for NPS of dementia

Study/author	Sample (n) age (years)	Diagnosis and staging	Compound and therapeutic scheme	Overall efficacy and outcome	Safety and tolerability	Comments
Controlled clinical trials (evidence level = 1)						
Volicer et al.^17^	11 (72.7)	AD mostly severe dementia	Crossover, two arms: dronabinol 2.5 mg/day fixed dose (6 weeks) + placebo (6 weeks). Placebo (6 weeks) + dronabinol 2.5 mg/day fixed dose (6 weeks).	Improvement of agitation and anorexia	Mild euphoria; somnolence, tiredness; seizure (n = 1)	Seizure was the most relevant adverse event (n = 1); causality unclear (dronabinol or disease progression); effect size not mentioned; p < 0.0005 for agitation (CMAI) and anorexia.
Mahlberg et al.^18^	24 (79.0)	AD severe dementia	Two intervention groups vs. placebo for 2 weeks. Dronabinol 2.5 mg (n = 7), melatonin 3.0 mg (n = 7) or placebo (n = 10).	Both treatment groups exhibited improvement of nocturnal agitation as measured by actigraphy	Not reported	Combined results of both intervention groups precludes identification of specific side effects from dronabinol; effect size not mentioned; p = 0.033 for agitation measured by NPI; p < 0.05 for actigraphic nocturnal activity.
van den Elsen et al.^19^	22 (76.4)	AD, VD, AD/VD severe dementia	Δ9-THC formulation low- and high dose; equivalent to 0.75 mg or 1.5 mg/day of dronabinol vs. placebo for 12 weeks.	No overall benefits for NPS, including agitation and aggression	Well tolerated and safe	Relatively mild NPS at baseline may have reduced the chance of detecting potential changes as outcomes; effect size not mentioned; p = 0.51 and 0.22 for NPI, CMAI, and ZBI.
van den Elsen et al.^20^	50 dronabinol: 24 (79.0) placebo: 26 (78.0)	AD, VD, AD/VD severe dementia	Δ9-THC formulation (fixed-dose dronabinol 1.5 mg t.i.d. for 3 weeks)	No benefits in agitation or aggression	Well tolerated and safe	Behavioral improvements in the placebo group possibly due to improved care during the trial; effect size not mentioned; p > 0.05 for NPI, CMAI, Barthel Index, CCGIC, QoL-AD.
Herrmann et al.^21^	38 (87.0)	AD moderate and severe dementia	Randomized, double-blind, crossover design: nabilone 1.0-2.0 mg/day for 6 weeks; placebo for 6 weeks; 1-week washout between interventions.	Decrease of agitation and reduction of caregiver burden	Sedation	Patients had resistant agitation, with inadequate response to psychotropics; effect size = 0.52, representing a medium effect; p = 0.003 for agitation (CMAI) and p = 0.001 for agitation (NPI); p *=* 0.009 for CGIC.
Open-label and observational studies (evidence level = 2)						
Walther et al.^22^	6 (81.5)	AD, VD severe dementia	Δ9-THC (dronabinol) 2.5 mg/day for 2 weeks	Improvement of agitation, aberrant motor behavior, nighttime behaviors, irritability, and appetite disorders	Well tolerated	No significant changes in psychotic symptoms (delusions, hallucinations) or apathy; effect size not mentioned; p < 0.05 for nocturnal motor activity (wrist actometer); p = 0.027 for NPI.
Shelef et al.^23^	10 (73.2)	AD moderate to severe dementia	Medical cannabis oil containing THC extract and phytocannabinoids; 2.5-5.0 mg/day (7.5 mg/day subsequently if well tolerated) for 4 weeks.	Improvement of agitation, aggression, aberrant motor behavior, delusions, sleep disorder, nighttime behavior, irritability, and apathy; decrease of caregiver distress.	Dysphagia, confusion, and falls observed in three patients	One patient had dysphagia and discontinued the treatment, another had recurrent falls, and a third patient became delirious with higher doses, but improved upon reduction (from 5.0 mg/day b.i.d. to 2.5 mg/day b.i.d.); effect size not mentioned; p < 0.05 to < 0.01 for NPI.
Broers et al.^24^	10 (79.5), all female	AD, VD, AD/VD	THC/CBD-based oil preparation 7.6 mg/13.2 mg (respect.) for 2 weeks), followed by 9.0 mg/18.0 mg for 2 months); continuation phase thereafter.	Improvement in global NPS, particularly, agitation; in motor rigidity, and performance of daily activities; patients became calmer and smiling more.	Acceptable tolerability	One particular feature of the study concerns the fact the authors prescribed natural cannabis extract, with a THC/CBD ratio of 1:2; unlike other studies, this used higher THC doses (9.0 mg vs. 5.0-7.0 mg/day); effect size not mentioned; no statistical analyses; only descriptive results
Case reports (evidence level = 3)						
Passmore^16^	1 (72.0) male	AD severe dementia	Nabilone 0.5 mg b.i.d. for 6 weeks	Improvement in agitation and aggressive behavior	Well tolerated and safe	After 6 weeks of substantial global improvement, NPS remained controlled for the following 3 months; effect size not mentioned; no statistical analyses.
Walther et al.^25^	2 (patient A, 75.0; patient B, 81.0)	AD moderate dementia	Crossover, 2 weeks: A: dronabinol 2.5 mg/day followed by placebo; B: placebo followed by dronabinol 2.5 mg/day.	Patient A: improvement in nighttime agitation; patient B improved only in the first week of dronabinol treatment.	Well tolerated	Short-lived or controversial benefits, but as dronabinol was well tolerated, the authors suggested new trials with higher dosages; effect size not mentioned; no statistical analyses.
Amanullah et al.^26^	1 (79.0) male (case A)	AD severe dementia	Nabilone 0.5 mg q.d. up to 0.5 mg t.i.d. (1 week); extended period up to day 78.	Decreased psychomotor agitation and aggression, including skin breakdown	Well tolerated	Nabilone alone may not be sufficient to control agitation since other drugs continued to be necessary; however, nabilone contributed to reducing doses of other antipsychotic drugs; effect size not mentioned; no statistical analyses.
	1 (60.0) male (case B)	FTD severe dementia	Nabilone 0.5 mg q.d. up to 0.5 mg t.i.d. (1 week); extended period up to day 63.	Decreasing agitation and increasing cooperation with personal care; starting to smile; better communication.	Well tolerated	
Zajac et al.^27^	1 (71.0)	FTD severe dementia	Nabilone 0.5-1.0 mg b.i.d. for 14 days; resumed after discontinuation and maintained for 3 months.	Improvement in sexual disturbances in the initial phase; relapse upon discontinuation; sustained improvement with reintroduction of treatment.	Sedation, lethargy, and delirium probably caused by drug interactions	The brief period of sedation, lethargy, and delirium at the beginning of treatment may have been a result of side effects precipitated by an increased dosage of nabilone to 1.0 mg twice a day plus interaction with other medications, e.g., lorazepam and risperidone required to control behavioral disturbances; effect size not mentioned; no statistical analyses.
Wilhelm et al.^28^	1 (78.0) male	LBD severe dementia	Dronabinol 2.5 mg/day to 12.0 mg/day	No effect at lower doses; worsening of agitation at higher doses.	Worsening of agitation	Improved only after adding dextromethorphan; effect size not mentioned; no statistical analyses.
Defrancesco and Hofer^29^	1 (79.0) female	AD severe dementia	Dronabinol (magistral formulation) equivalent to 4.9-6.7 mg/day for 8 months	Improvement in agitation, disruptive behavior, aggression, and anxiety	Well tolerated, no side effects	Benefits of dronabinol for aggression and anxiety are mediated by activation of CB1 and activation of endocannabinoids; effect size not mentioned; no statistical analyses.
Gopalakrishna et al.^30^	3 (63.0, 65.0, and 69.0) all female	FTD severe dementia	CBD (63 years) Medical marijuana (65 and 69 years)	Improvement of mood and impulsive/intrusive behavior (CBD); improvement of anxiety (medical marijuana).	No side effects reported	Patient on CBD had vivid, unpleasant dreams which improved with addition of THC; effect size not mentioned; no statistical analyses.

AD = Alzheimer’s disease; AD/VD = mixed AD/vascular dementia (VD); b.i.d. = *bis in die* (twice a day); CB1 = cannabinoid receptor type 1; CBD = cannabidiol; CCGIC = caregiver’s clinical global impression of change; CMAI = Cohen Mansfield Agitation Inventory; NPI = Neuropsychiatric Inventory; FTD = frontotemporal dementia; LBD = Lewy body dementia; NPS = neuropsychiatric symptoms; q.d. = *quaque die* (once a day); (QoL-AD = Quality of Life–Alzheimer’s Disease Scale; THC = Δ^9^-tetrahydrocannabinol; t.i.d. = *ter in die* (three times a day); ZBI = Zarit Burden Interview.

The studies were also summarized according to the level of evidence supporting the effects of the various cannabinoid drugs for treatment of NPS ( Table 2 ). Considering all selected publications, only one randomized-controlled trial (RCT) reported the effect size (0.51; a medium effect; Herrmann et al.^21^ ); and in general, the statistical significance related to outcomes of studies varied widely.

**Table 2:** NPS and drug efficacy

Drug/diagnosis	Study design (authors)	Efficacy for NPS	Level of evidence
Dronabinol			
AD	Controlled trial (Volicer et al.^17^)	Improvement in agitation and aggression and anorexia	1
AD	Controlled trial (Mahlberg et al.^18^)	Improvement of nocturnal agitation measured by actigraphy	1
AD, VD, AD/VD	Controlled trial (van den Elsen et al.^19^)	No benefits	1
AD, VD, AD/VD	Controlled trial (van den Elsen et al.^20^)	No benefits	1
AD, VD	Open-label (Walther et al.^22^)	Improvement in agitation, aggression, nighttime behaviors, irritability, and appetite disorders	2
AD	Case report (Walther et al.^25^)	Improvement in nighttime agitation (patient B: short-lived benefits)	3
AD	Case report (Defrancesco and Hofer^29^)	Improvement in agitation, behavioral disruption, aggression, and anxiety	3
LBD	Case report (Wilhelm et al.^28^)	Worsening of agitation	3
AD	Controlled trial (Herrmann et al. ^21^)	Improvement in agitation and caregiver burden	1
Nabilone			
AD (A), FTD (B)	Case report (2) (Amanullah et al.^26^) (case A and B)	A) Improvement in agitation and aggression B) Improvement in agitation	3
AD	Case report (Passmore^16^)	Improvement in agitation and aggression	3
FTD	Case report (Zajac et al.^27^)	Improvement in sexual disorders	3
AD	Open-label (Shelef et al.^23^)	Improvement in agitation, aggression, delusions, nighttime behavior, apathy, and caregiver distress	2
Medical cannabis oil			
AD, VD, AD/VD	Open-label (Broers et al.^24^)	Improvement in agitation and motor rigidity	2
THC/CBD-formulation			
FTD	Case report (3) (Gopalakrishna et al.^30^)	Improvement in agitation and anxiety	3

Levels of evidence: 1 = controlled clinical trial with placebo; 2 = open-label or observational study, without placebo; 3 = case report.

AD = Alzheimer’s disease; AD/VD = mixed AD/vascular dementia (VD); CBD = cannabidiol; FTD = frontotemporal dementia; LBD = Lewy body dementia; NPS = neuropsychiatric symptoms; THC = Δ^9^-tetrahydrocannabinol.

## Discussion

In spite of the enthusiasm of many clinicians towards use of medical cannabinoids for treatment of agitation and other NPS in dementia, there is still very limited evidence from controlled studies to support this prescription.^32 , 33^ These compounds, including magistral formulations of marijuana extracts and pharmaceutical products containing THC and/or CBD, have been tentatively used to treat difficult cases of NPS/BPSD where more established pharmacological and non-pharmacological strategies had failed to deliver benefits.^23 , 34^ Controversial results from controlled trials, along with a limited number of successful clinical experiences, have been reported in the format of case reports or small case series.^34^ Although clinically relevant, these communications presumably represent a collection of cases where the use of cannabinoid drugs yielded positive responses, amidst a probably larger number of failed attempts – illustrating the burden of publication bias.^34^ Nonetheless, the available studies – controlled or not – substantiate the case for further experimentation and emphasize the urgent need for randomized, placebo-controlled trials conducted with larger and better selected patient samples.^32^ There is also need for better characterization of the behavioral symptoms that may be most responsive to the effects of cannabinoid drugs, in addition to detailed profiling of compounds, doses, and treatment duration.^33 , 35^

In the present study, we analyzed the available publications that were systematically retrieved from the literature according to the criteria established, using MeSH terms clustered into three classificatory domains, i.e., “cognitive impairment/dementia,” “NPS/BPSD,” and “cannabinoid drugs.” The total number of studies reporting original clinical data about use of medical cannabinoids for treatment of NPS was small (n = 15), compared to a 50-fold larger number of publications on this very topic, which obviously include pre-clinical and neurobiological studies, reviews of the literature, and position papers disclosing expert points of view. We further subdivided the eligible papers into three categories that reflect the level of evidence embedded in these contributions, i.e., controlled clinical trials, open-label or observational studies, and case reports. These selected publications comprised five controlled trials, three open-label/observational studies, and seven case reports, which represent the body of publications on this subject for the time being – not much, but valuable. The level of evidence provided by the different studies will be discussed across the topics “efficacy,” “safety/tolerability,” and “limitations.”

### Efficacy

Controlled clinical trials (level of evidence 1) with dronabinol (Mahlberg et al.,^18^ ) and nabilone^21^ (Herrmann et al.^21^ ) demonstrated a consistent improvement in agitation, nighttime behavior, and aggression in patients with severe AD. Open-label studies (level of evidence 2) with dronabinol,^22^ MCO,^23^ and THC/CBD-based medicine^24^ also showed efficacy in controlling agitation and other behavioral disturbances in patients with severe AD, VD, and AD/VD. Broers et al.^24^ prescribed natural cannabis extract, with a THC/CBD ratio of 1:2, and this was a particular feature of their study. In contrast to other authors, they ordered higher dosages, with an average of 9.0 mg THC, while most studies recommended lower dosages, from 2.0 mg to 7.0 mg.^36^ Broers et al.^24^ recognized some weaknesses of their study given its observational design.

Likewise, medical cannabis even provoked substantial improvement in delusions and apathy in moderate and severe AD, with subsequent reduction in caregiver distress, according to an open-label study (level of evidence 2).^23^ Therefore, case reports (level of evidence 3) revealed improvement with dronabinol in distinct neuropsychiatric domains in severe AD, including agitation and nighttime behaviors,^25 , 29^ as well as in agitation and aggression with nabilone.^16 , 26^

Notably, in patients with severe FTD, nabilone was effective for control of agitation and aggression,^26^ with reduced disinhibition and sexual disorders.^27^ Favorable results were also observed in patients with FTD suffering from agitation and anxiety who were given CBD (medical marijuana).^30^

Conversely, in two controlled trials (level of evidence 1), enrolling patients with severe AD, VD, and AD/VD, who were suffering from agitation and other NPS, the authors found no significant differences between dronabinol and placebo.^19 , 20^ Moreover, one of the patients (“B”) with severe AD^25^ who received dronabinol, achieved an improvement in nighttime agitation for only a very short period. Additionally, two clinical trials with this drug showed no benefits for NPS. Along the same lines, a patient with LBD and resistant aggressive behavior had no response to dronabinol, and actually got even worse with the treatment.^28^

Woodward et al.^37^ conducted a retrospective analysis based on medical records in order to detect the efficacy and tolerability of dronabinol for treatment of inpatients with severe dementia from different etiologies (AD, VD, AD/VD, and FTD). Clinically relevant behavioral disturbances, e.g., agitation, aggression, sleep disorders, and anorexia were the main focus of analyses. The authors concluded that dronabinol may be an adjunctive strategy for treatment of NPS in dementia. It is plausible to draw attention to favorable effects of medical cannabinoids on regulation of circadian rhythm and sleep architecture, as well as in controlling nocturnal behaviors of patients with advanced stages of AD.^38 , 39^

According to Murillo-Rodriguez,^31^ the effects of dronabinol on circadian rhythm can be related to the eCB, which modulates sleep related-properties via CB1 receptors in the brainstem. Furthermore, experimental investigations represent a basis for new clinical trials aimed at expanding knowledge about the potential benefits of medical cannabinoids. Notably, THC decreases agonistic acts in multiple mammalian species (mice, rats, and squirrel monkeys) undergoing intruder confrontation.^40 , 41^ Mutant mice lacking CB1 exhibit more aggressive or defensive behavior, such as avoidance, freezing, and risk-assessment actions, suggesting CB1 plays a role in buffering against social stress.^42 , 43^ The statistical significance varied widely between studies in our analysis, both according to the results obtained by different assessments, as well as within the same scale when measuring selected psychopathological domains.

Concerning NPS related to different entities in other populations, medical cannabinoids have also shown benefits. For instance, according to a review on CBD treatment of non-motor symptoms of Parkinson’s disease, patients who received this compound exhibited significant improvement in psychosis and sleep behavior disorder, as well as better performance in daily activities, and became more able to deal with the stigma related to the disease.^44^ Furthermore, patients with severe treatment-resistant epilepsy for whom CBD was prescribed in addition to antiepileptic drugs, exhibited an improvement in behavioral disorders and in quality of life and also achieved a reduced frequency of seizures.^45^ An important methodological weakness of studies carried out in different populations, including our investigation, concerns the small sample size and short-term interventions. Additionally, a considerable number of clinical studies on medical cannabinoids for treatment of NPS in dementia and other clinical entities, have failed or have not reached a decisive agreement on their effectiveness.^19 , 20 , 28 , 33 , 34 , 46^

### Safety and tolerability

Regarding safety and tolerability, participants in general presented mild side effects with dronabinol, nabilone, and other cannabinoid compounds. Dronabinol and nabilone can cause transient sedation, somnolence, and mild euphoria, possibly related to interaction with basic-routine drugs. One patient presented lethargy and delirium during nabilone use in the context of polypharmacy, which at least in part could explain this event. Another one had a generalized seizure during dronabinol use, but it was not clear whether the event was caused by the drug or by progression of the AD pathology itself. In this context, Zajac et al.^27^ emphasized the risk of sedation, lethargy, and delirium in patients with dementia when prescribed medical cannabinoids. Woodward et al.^37^ observed that such events either occurred amidst pharmacological interactions with other drugs in use, or could be related to general medical comorbidities. Defrancesco and Hofer^29^ draw attention to the fact that the hepatic metabolism of THC by cytochrome P450 isoenzymes can cause a considerable risk of drug-drug interactions. Although in general cannabinoids are well tolerated, it is mandatory to monitor potential side effects given the marked vulnerability of patients with dementia.

The safety and tolerability of medical cannabinoids has also been the subject of a recent comprehensive review of randomized controlled trials covering populations other than those with dementia from different etiologies.^47^ The review included patients with Parkinson’s disease, multiple sclerosis, Huntington disease, advanced cancer with pain, rheumatoid arthritis, and diabetes, among others. The authors reported no significant increase in serious events and recognized medical cannabinoids in general as a safe and relatively well-tolerated treatment in older adults.^47^ Likewise, according to a review conducted by Crippa et al.^44^ in patients with Parkinson’s disease, CBD was well tolerated when prescribed for treatment of non-motor symptoms.

### Methodological aspects and limitations

RCTs with adequate sample sizes, reputed as the best approach to validate new therapeutic interventions, are still lacking in this field of knowledge. To date, only six controlled studies have been conducted to test the efficacy and safety of medical cannabinoid drugs for treatment of NPS/BPSD, jeopardizing construction of a solid evidence-based background (level of evidence 1). In addition, in many such studies, the patient samples were small, with fifty participants or less. Three studies had an open-label design (level 2), also with small sample sizes ranging from six to 10 subjects. By definition, case reports (level 3) enrolled even smaller numbers of patients. Heterogeneity of samples is another important issue, since most of the aforementioned studies comprised patients with dementia of different etiologies (e.g., AD, VD, AD/VD, FTD, and DLB), with distinct profiles of neurobehavioral disturbances. Such variability within groups may have impacted on outcomes and responses to treatment, restricting the comparability of studies and extrapolation of findings. Such methodological characteristics have also been pointed out by previous review studies on NPS in older populations.^34 , 46 , 47^

It is also worth mentioning that controlled trials and open-label studies were characterized by different periods of intervention, extending from 2 to 14 weeks. Case studies also varied widely, from 7 to 78 days. Likewise, the assessment of outcomes differs from study to study, most lacking more comprehensive and longitudinal appraisals. Most patients were classified as having advanced dementia, and a smaller group presented moderate dementia. These data are clinically relevant because NPS, especially agitation, nighttime behavior, wandering, sleep disorders, eating disorders, sexual disinhibition, and apathy tend to worsen in the more advanced stages of dementia, increasing caregiver burden.^33 , 48 , 49^ Another aspect to be considered regards variation in the dosage regimens of different compounds. Some studies adopted fixed doses of the cannabinoid drugs, while others used variable prescriptions throughout the investigation. This fact suggests that the therapeutic window for this class of drugs is yet to be defined. Notably, the dosing schedule for medical cannabinoids aims to determine the best efficacy of the compound and, at the same time, maintain an acceptable level of possible adverse events.^50^

Several aspects were related to the decision to restrict our study to a systematic review without a meta-analysis of primary outcomes. Factors that precluded the meta-analytical approach were: incomplete data reported in the original manuscripts, heterogeneous methodological designs, different drug dosages, different delivery routes adopted for therapy, variation in the percentage of pharmacological compounds of medical cannabinoids, and different periods of treatment. Another limitation of our study is that we did not register the review and a protocol was not prepared in advance. Further trials incorporating methodological designs based on larger and longer-term investigations are strongly advocated to determine the real-world safety and effectiveness of medical cannabinoids. Studies should establish an appropriate dosage-escalation regimen to maximize efficacy while at the same time keeping adverse events acceptable, in particular for fragile patients with associated comorbidities. Another critical point concerns explanation of neurobiological mechanisms and the pharmacological actions of cannabinoids within the brain that are involved in behavior regulation.

## Conclusion

Cannabinoid-based drugs are a promising psychopharmacological strategy to treat patients with NPS related to moderated or advanced dementia. In general, studies with medical cannabinoids have shown favorable results, although some do not provide convincing efficacy. Basically, compounds are well tolerated, but given the vulnerability of patients with dementia they require appropriate monitoring by the clinician. Development of controlled trials with longitudinal design and larger samples is needed to determine their long-term efficacy, adverse events, risks of interactions with other drugs, and respective dosage ranges, as well as the most suitable therapy period. Within this approach, determining the respective THC/CBD concentrations for specific drugs to treat different psychopathological domains in dementia still remains an additional concern.
